# Hardware in the Loop Performance Assessment of LIDAR-Based Spacecraft Pose Determination

**DOI:** 10.3390/s17102197

**Published:** 2017-09-24

**Authors:** Roberto Opromolla, Giancarmine Fasano, Giancarlo Rufino, Michele Grassi

**Affiliations:** Department of Industrial Engineering, University of Naples “Federico II”, P.le Tecchio 80, 80125 Naples, Italy; giancarmine.fasano@unina.it (G.F.); giancarlo.rufino@unina.it (G.R.); michele.grassi@unina.it (M.G.)

**Keywords:** spacecraft pose determination, uncooperative targets, LIDAR, monocular camera, LIDAR/camera relative calibration, hardware-in-the-loop laboratory tests

## Abstract

In this paper an original, easy to reproduce, semi-analytic calibration approach is developed for hardware-in-the-loop performance assessment of pose determination algorithms processing point cloud data, collected by imaging a non-cooperative target with LIDARs. The laboratory setup includes a scanning LIDAR, a monocular camera, a scaled-replica of a satellite-like target, and a set of calibration tools. The point clouds are processed by uncooperative model-based algorithms to estimate the target relative position and attitude with respect to the LIDAR. Target images, acquired by a monocular camera operated simultaneously with the LIDAR, are processed applying standard solutions to the Perspective-*n*-Points problem to get high-accuracy pose estimates which can be used as a benchmark to evaluate the accuracy attained by the LIDAR-based techniques. To this aim, a precise knowledge of the extrinsic relative calibration between the camera and the LIDAR is essential, and it is obtained by implementing an original calibration approach which does not need ad-hoc homologous targets (e.g., retro-reflectors) easily recognizable by the two sensors. The pose determination techniques investigated by this work are of interest to space applications involving close-proximity maneuvers between non-cooperative platforms, e.g., on-orbit servicing and active debris removal.

## 1. Introduction

Pose determination is a very important task for space activities like On-Orbit Servicing (OOS) and Active Debris Removal (ADR), in which a main spacecraft (commonly called chaser) has to approach a man-made target (e.g., operative/inoperative satellites or abandoned rocket bodies) to carry out inspection, repair, maintenance or refueling operations [1], as well as safe disposal [2]. Among these activities, autonomous refueling is receiving increasing attention due to its promising economic return [3]. A review of existing and future concepts for space refueling with an analysis of the associated risks can be found in [4]. Besides spacecraft proximity operations, another mission scenario for which pose determination techniques play a key role is planetary exploration. In fact, autonomous landing on unknown environment must rely on robust terrain-relative navigation algorithms. In this respect, LIDAR represent a promising solution [5].

Focusing on spacecraft proximity operations, the target pose, i.e., the set of parameters describing its relative position and attitude with respect to the chaser, can be used to reconstruct the entire relative navigation state (including the relative translational and angular velocities) by exploiting adequate filtering architectures [6]. This information is necessary to plan and safely execute close-proximity maneuvers on orbit, like rendezvous, fly-around and docking.

In the case of OOS and ADR scenarios, the target does not have a communication link to exchange information with the chaser and it is not equipped with antennas or optical markers which can be used to cooperatively attain high-accuracy estimates of relative position and attitude using radio-frequency (RF) [7,8], satellite navigation (GNSS) [9], or electro-optical (EO) technologies [10,11]. Such targets are classified as non-cooperative, and pose determination can be achieved by relying exclusively on the data collected by EO sensors, namely active LIDARs operating in the near-infrared band, or passive monocular and stereo cameras operating in the visible band. Passive infrared cameras, instead, do not represent a valuable option since they allow target line-of-sight estimation at far range, but their images are too poorly textured for full pose determination purposes, when acquired in close-proximity [12].

A trade-off between active and passive EO sensors should be done considering the corresponding advantages and drawbacks in terms of type of output data, mass, cost, power consumption, and robustness against variable illumination conditions [13]. In this respect, LIDAR-based solutions are investigated in this work, due to the capability to operate even in dark environment and to provide 3D representations of the target (i.e., point clouds). Indeed, stereo cameras have similar 3D imaging capabilities but they are passive sensors and their operative range and range-measurement accuracy are negatively affected by the length of the stereo baseline which is limited by the size of the chaser. Also, attention is addressed to the task of pose initial acquisition, that is performed with no prior knowledge about the target relative position and attitude.

If the target geometry is known (at least coarsely), the pose parameters can be estimated by trying to optimize the match between the acquired data and a target model (built either off-line or directly on board). Many model-based approaches have been recently proposed in the literature. Most of them are designed to operate on raw data, i.e., point-based algorithms [14,15,16,17,18], while others rely on the extraction of natural features or more complex descriptors, i.e., feature-based algorithms [19,20]. The results presented in these works highlight that some open challenges still need to be addressed. Indeed, the computational effort shall be restrained when dealing with fast relative dynamics, and the algorithms’ robustness shall be demonstrated against highly variable relative position and attitude states [21].

In this context, two original point-based algorithms, which exploit the concepts of point-cloud centroiding, Template Matching (TM) [22] and Principal Component Analysis (PCA) [23], have been recently developed by the Aerospace Systems Team of the University of Naples "Federico II" for pose initial acquisition of a non-cooperative spacecraft. These algorithms have been designed to be robust against pose variability as well as to optimize computational efficiency while simultaneously restraining the amount of on-board data storage. This latter point has been made possible by the dynamic (i.e., on-board) generation of a database of templates, which means without requiring any complex off-line learning phase. The pose determination performance of these techniques, namely the on-line TM [16] and the PCA-based on-line TM [18], has been assessed by means of numerical simulations, carried out within a virtual environment designed to realistically reproduce LIDAR operation and target/chaser relative dynamics. Simulation results have proved that the two algorithms are able to provide fast and reliable initial pose estimates for a wide range of relative attitude and distances and considering targets characterized by different size and shape [16,18]. In this respect, numerical analyses ensure much larger flexibility than experimental tests. However, despite the scalability issues inherent to the design of a laboratory facility (especially in terms of power budget of the emitted and backscattered laser shots and target/chaser size and geometry) and the difficulty of realistically reproducing space imaging conditions [24], experimental analyses have critical importance to validate and complement numerical results. Indeed, on one side, they allow evaluating the effect on algorithms’ performance of those noise sources in the LIDAR measurements which are difficult to be consistently included in the generation of synthetic datasets. On the other side, they can reproduce imaging conditions of low contrast between the target and the background (which is also hard to obtain realistically through numerical simulations). Hence, the goal of this work is to describe the experimental strategy adopted to evaluate both effectiveness and accuracy of the on-line TM and the PCA-based on-line TM algorithms using point-cloud data collected by a 3D scanning LIDAR exploiting the Time-of-Flight (TOF) measurement principle.

Clearly, an independent, high-accuracy estimate of the relative position and attitude of the target with respect to the sensor should also be obtained to get a benchmark for performance assessment of the above-mentioned LIDAR-based techniques. Marker-based motion capture systems can provide positioning and attitude accuracy of sub-millimeter and cents-of-degree order, respectively [25]. However, they rely on both expensive hardware (i.e., multiple, high-resolution cameras) and processing software. Here, a cost-effective and simple solution to obtain a benchmark pose estimate is adopted. Specifically, a single monocular camera is used to image a set of cooperative markers, i.e., a planar checkerboard grid, attached to the target surface, and the acquired image is processed implementing a standard solution to the Perspective-*n*-Point (P*n*P) problem [26].

It is now worth outlining that this test strategy can be effective only if the LIDAR/camera extrinsic calibration, i.e., the relative position and attitude between the camera and the LIDAR, is carried out accurately. This task has been recently addressed in the literature when dealing with 2D [27,28,29,30] and 3D [31,32,33] scanning LIDARs. These approaches are based on the iterative minimization of a non-linear cost function in a least-squares sense. Focusing on the case of 3D LIDARs, a planar calibration target is adopted in [31,32]. Instead, a target-less method for the extrinsic calibration between a 3D LIDAR and an omnidirectional camera is presented in [33], which is based on the registration of mutual intensity and reflectivity information from the camera and laser data. In the above-mentioned works a revolving-head 3D LIDAR is considered. In this paper, an original semi-analytical approach is presented for the relative calibration between a 3D scanning LIDAR (having a fixed optical head and internal rotating mirrors) and a monocular camera. The main innovative aspects of this method can be listed as follows: (1) It is non-iterative; (2) It relies on basic (i.e., easy to satisfy) geometric constraints; (3) It allows finding homologous points between the camera images and the LIDAR point clouds without the need of ad-hoc markers (e.g., retro-reflectors) easily detectable and recognizable in the acquired data; (4) This approach has also the advantage of being easily applicable by exploiting dedicated tools in MATLAB, namely the camera calibration Toolbox [34] and the Curve Fitting Toolbox [35].

The paper content is organized as follows: Section 2 describes the elements composing the experimental setup and the adopted test strategy. Section 3 presents the LIDAR- and camera-based pose determination algorithms. Section 4 illustrates the LIDAR/camera relative calibration procedure. Finally, Section 5 includes the results of the pose determination experiments, while Section 6 draws the conclusion.

## 2. Experimental Setup: Description and Test Strategy

**Figure 1:**
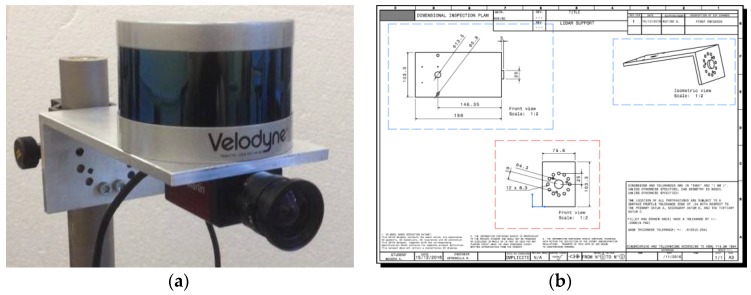
LIDAR/camera installation (**a**). Technical drawing of the L-shaped aluminum bracket (**b**).

The main elements composing the experimental setup are the EO sensors adopted for monocular- and LIDAR-based pose determination, the target and a set of calibration tools. With regards to the pose determination sensors, a 3D scanning LIDAR, namely the VLP-16 produced by Velodyne (San Jose, CA, USA) [36], and a monocular camera, namely the AVT Marlin F-145C2 produced by Allied Vision Technologies (AVT, Newburyport, MA, USA) [37] are adopted. The VLP-16 is a Class 1 laser product (eye safe) operating in the near infrared (900-nm wavelength) and exploiting the TOF principle to get distance measurements. This sensor scans a 360°-Field-of-View (FOV) in azimuth with resolution of 0.1° by rotating a set of 16 laser/detector pairs mounted in a compact housing. These laser sources are vertically separated by 2°, thus covering a 30°-FOV in elevation. Hence, the target point cloud is highly sparse (i.e., it has very low density) in the vertical direction. This aspect is advantageous in terms of the reduced amount of data to be processed (and, consequently, the computational load). However, it also makes the pose determination task more challenging since very limited target details are visible. Indeed, ambiguities may arise and high-accuracy pose estimates are difficult to obtain. On the other hand, the AVT camera images a 48.5° × 37.3° FOV on a 1280 × 960 pixel detector, thus ensuring a much higher spatial resolution, i.e., 0.04° both in the vertical and horizontal directions. The two sensors are rigidly mounted on an aluminum bracket, an L-shaped support which ensures a fixed relative configuration to be kept. The bracket is designed so that the sensors’ boresight axes are nearly co-aligned, thus maximizing the portion of the space shared by their FOVs. The bracket is also the mechanical interface to install the two sensors on a vertically-adjustable post (a StableRod by Melles Griot, Carlsbad, CA, USA), fixed on the optical table which firmly supports the whole laboratory test system. This installation is in Figure 1, which also shows the technical drawing of the bracket.

**Figure 2:**
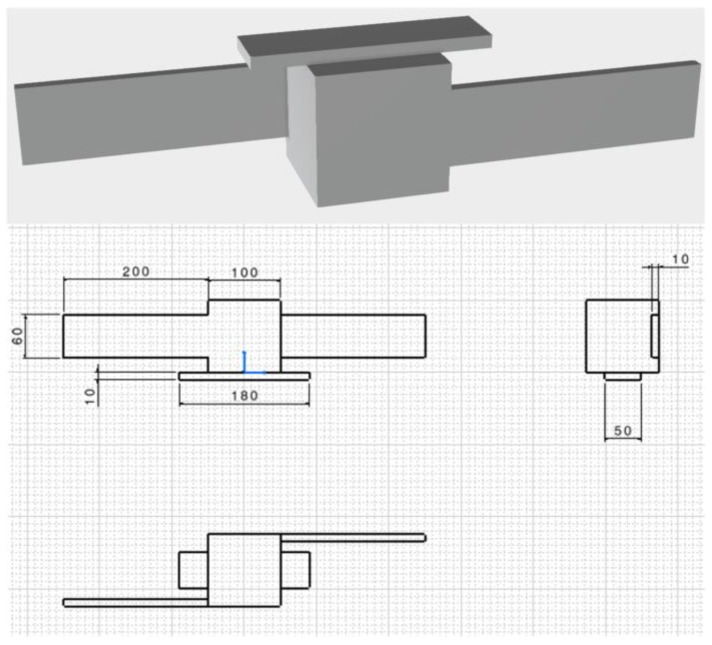
CAD representation of the satellite mock-up (**up**). Technical drawings (dimensions are in mm) (**down**).

The target is a scaled replica of a LEO satellite, composed of a rectangular antenna and two solar panels attached to the surfaces of a cuboid-shaped main body. This simplified geometry, inspired by the satellites composing the COSMO-SkyMed constellation, is 3D printed on polylactic acid. The mock-up dimensions, i.e., 0.5 m × 0.1 m × 0.1 m, as well as the range of distances considered for the pose determination tests, i.e., from 0.5 m up to 1.8 m, are selected in view of the limited size of the optical bench hosting the experimental setup, i.e., 2 m × 1.25 m. While the angles are scale-invariant, meaning that the relative orientation between the sensor and the target is unaffected by the scale, the distances are linearly scaled with the size of the target [24]. Hence, the results provided by the experimental tests presented in this paper are valuable for a range of actual distances going from 15 m to 54 m, considering that the target scale is approximately 1/30 compared to the actual size of COSMO-SkyMed-like satellites. This separation range is relevant to a close-proximity flight condition in which the chaser executes operations such as fly around and monitoring. A CAD representation of this satellite mock-up is shown in Figure 2 together with basic technical drawings.

Finally, two checkerboard grids are adopted as calibration tools. Specifically, a 40 cm × 28 cm checkerboard grid is attached on a planar support made of plexiglass. This calibration board is used for both the intrinsic calibration of the ATV camera and the LIDAR/camera extrinsic calibration. On the other hand, a 10 cm × 10 cm checkerboard grid is attached on one of the surfaces of the target main body to obtain a set of cooperative markers which are exploited by the monocular-based pose estimation algorithm.

**Figure 3:**
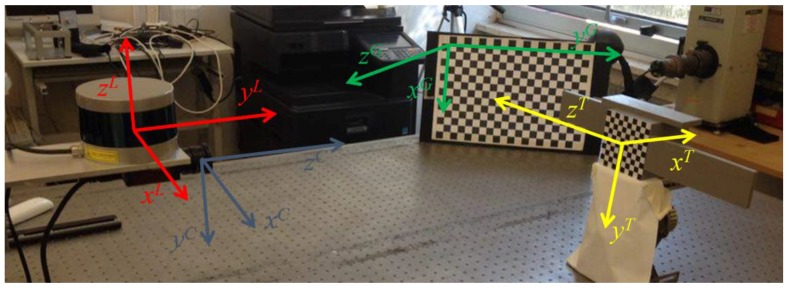
Experimental setup and reference frames definition.

An overall representation of the above-mentioned elements is given by Figure 3, where the four reference frames, required to describe the pose estimation algorithms and the relative calibration method, are also highlighted. The LIDAR reference frame (L) has the origin at the optical center; *y^L^* is the sensor boresight axis (i.e., corresponding to 0° in azimuth and elevation), *z^L^* is the scanning rotation axis and *x^L^* is pointed rightward to obtain a right-handed coordinate system. The camera reference frame (C) has the origin at the focal point; *z^C^* is directed along boresight, while the remaining axes, i.e., *x^C^* and *y^C^*, lie on the focal plane. Following the definition provided by the MATLAB Camera Calibration Toolbox, the reference frame of the rectangular checkerboard grid (G) has the origin at the upper-left corner [34]; *z^G^* is the outer normal to the plane, while *x^G^* and *y^G^* lie in the plane of the checkerboard. Finally, the Target reference frame (T) is located at the center of the main body; *z^T^* is directed along the solar panels, *y^T^* is directed as the inner normal to the antenna and *x^T^* completes a right-handed coordinate system.

**Figure 4:**
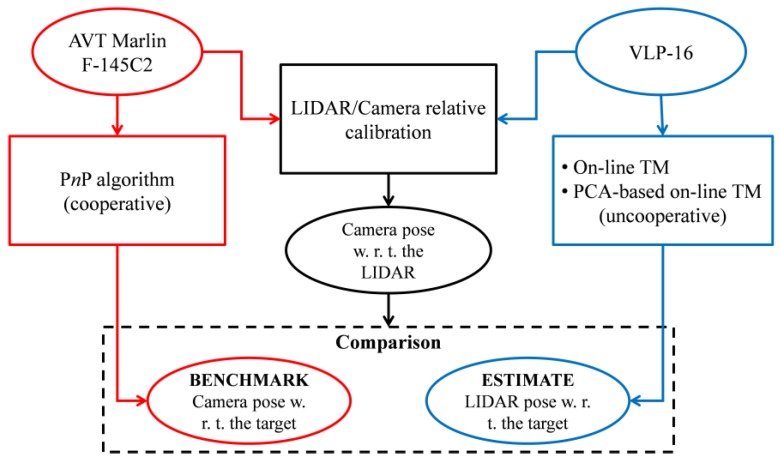
Test strategy conceived to evaluate the pose estimation accuracy of LIDAR-based pose determination algorithms. Input/output boxes are elliptical. Processing boxes are squared.

This experimental setup is used to carry out the test strategy summarized by the block diagram in Figure 4, where the monocular and LIDAR pose determination tests can be done only after the LIDAR/Camera relative calibration is completed.

The LIDAR and the camera provide point clouds and images of the target (if located in the shared portion of their FOVs). These data are processed to obtain an estimate of the target pose with respect to the two sensors (see Section 3 for details on the algorithms). At this point, the results of the LIDAR/camera relative calibration (see Section 4 for the detailed procedure) can be used to compare the LIDAR-based pose estimate to the monocular one.

## 3. Pose Determination Algorithms

The following rules are adopted for the mathematical notation in this paper. Italic type is used for each symbol, except for reference frames indicated by normal-type capital letters. Vectors and matrixes are also identified by a single and double underline, respectively. With regards to the parameterization of the pose, the relative attitude and position between two reference frames, e.g., A and B, is represented by the position vector (*T_A-B_^A^*) and the rotation matrix ($\underline{\underline{R}}$*_A-B_*). Specifically, *T_A-B_^A^* is the position of B with respect to A, expressed in A. Instead, $\underline{\underline{R}}$*_A-B_* is the transformation which allows converting a vector from A to B. Other sets of parameters representing the attitude between two reference frames equivalently to the rotation matrix, i.e., equivalent Euler axis and angle, Euler angle sequence, and quaternion, are also mentioned in the paper. Definition and conversion between these attitude representations can be found in [38].

### 3.1. LIDAR-Based Uncooperative Techniques

With regards to pose determination tasks, template matching is the process of looking within a database of templates for the one which provides the best match when compared to the acquired dataset. This leads to a pose estimate since each template is a synthetic dataset corresponding to a specific set of relative position and attitude parameters, and it is generated by a simulator of the considered EO sensors. The database shall be built by adequately sampling the entire pose space, which is characterized by six (i.e., three rotational and three translational) Degrees-of-Freedom (DOF). Clearly, a full-pose (i.e., 6-DOF) database is composed of so much templates that the required computational effort may be not sustainable for autonomous, on-board operations.

The on-line TM and the PCA-based on-line TM, tested in this paper, operate on 3D datasets, i.e., point clouds, and are both designed to significantly restrain the number of templates by limiting the pose search to less than six DOFs. Although, a detailed description of these algorithms is provided in [16,18], the main concepts useful for the understanding of the experimental results are presented below. First, the centroid of the measured point cloud (*P*_0_) is computed, thus getting a coarse estimate of the relative position vector between the LIDAR and the target (*T_L-T_^L^*). This approach is called centroiding and is realized using Equation (1):

(1) $${\underline{P}}_{0}=\frac{1}{N}\sum_{i=1}^{N}\left[\begin{array}{c} x^{L}{}_{i}\\ y^{L}{}_{i}\\ z^{L}{}_{i} \end{array}\right]$$

where (*x^L^_i_*, *y^L^_i_*, *z^L^_i_*) are the coordinates in L of each element of the LIDAR point cloud, and *N* is the total number of measured points.

This operation reduces the TM search to a 3-DOF database identified only by the relative attitude parameters, e.g., a 321 sequence of Euler angles (*γ*, *β*, *α*). For the on-line TM, the TM search is done directly after centroiding. Instead, the PCA-based on-line TM further restrains the size of the database before performing the TM search. To this end, PCA is used to estimate the principal direction of the measured point cloud as the eigenvector corresponding to the maximum eigenvalue of the associated covariance matrix. Indeed, if the target has an elongated shape, very common to orbiting satellites and launcher upper stages, this quantity represents a coarse estimate of the direction of the main geometrical axis of the target. Thus, two rotational DOFs (*α*, *β*) are estimated and the TM search is limited to a 1-DOF database (i.e., the unresolved rotation around the target main axis, *γ*). For both the algorithms, the unique tunable parameter is the angular step (*Δ*) with which the relative attitude space is sampled. Of course, the lower is *Δ*, the larger is the number of templates and, consequently, the required computational effort.

The TM search for the best template is done using a correlation approach. Specifically, the correlation score is computed as the mean squared distance of template/LIDAR points associated according to the Nearest Neighbor (NN) logic [39]. This correlation score can be evaluated as shown by Equations (2) and (3) for the on-line TM and PCA-based on-line TM, respectively:

(2) $$C(\alpha ,\beta ,\gamma )=\frac{1}{N}\sum_{i=1}^{N}{\left|\left({\underline{P}}^{i}-{\underline{P}}_{0})-({\underline{P}}_{temp}^{i}(\alpha ,\beta ,\gamma )-{\underline{P}}_{0temp}(\alpha ,\beta ,\gamma )\right)\right|}^{2}$$

(3) $$C(\gamma )=\frac{1}{N}\sum_{i=1}^{N}{\left|\left({\underline{P}}^{i}-{\underline{P}}_{0})-({\underline{P}}_{temp}^{i}(\gamma )-{\underline{P}}_{0temp}(\gamma )\right)\right|}^{2}$$

In the above equations, *P^i^* and *P_temp_^i^* are the corresponding points in the acquired datasets and the template, respectively, while *P*_0*temp*_ is the centroid of the template.

**Figure 5:**
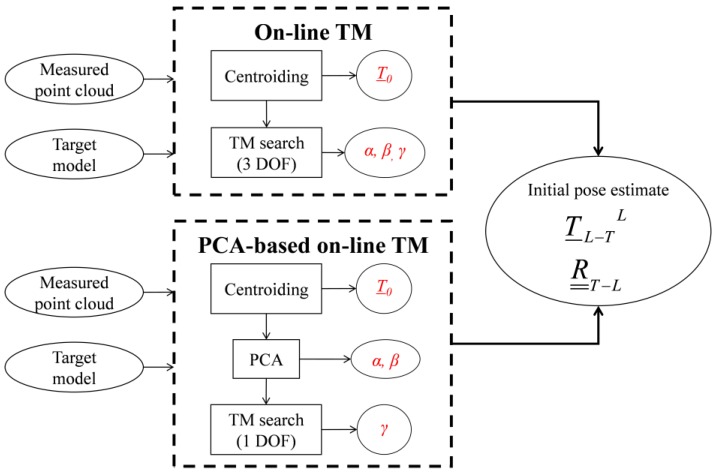
Flow diagram of the LIDAR-based algorithms for pose initial acquisition of an uncooperative target.

An overview of these algorithms is provided by the flow diagram in Figure 5. It can be noticed that they do not require any offline training stage with respect to the target model. Instead, the model is stored on board and the templates are built on line. The final output provided by both the algorithms is a coarse initial estimate of the relative position vector (*T_L-T_^L^*) and rotation matrix ($\underline{\underline{R}}$*_T-L_*).

### 3.2. Monocular Marker-Based Techniques

The pose of the camera with respect to the target is obtained by using a cooperative approach which is based on the determination of correspondences between 2D feature points extracted from the acquired image and 3D real points (markers) whose positions are exactly known in T. The cooperative markers are the corners of the checkerboard grid attached to the surface of the target. Once an image of the target is acquired, the first step of the pose determination process is the extraction of the corners of the checkerboard. Since the corner extraction process is driven by a human operator, this approach allows obtaining a set of 81 2D-to-3D (image/target) correct point matches. Given this input, the pose parameters are estimated solving the P*n*P problem. This means that a non-linear cost function, i.e., the re-projection error between real and image corners, is minimized in a least-squares sense.

**Figure 6:**
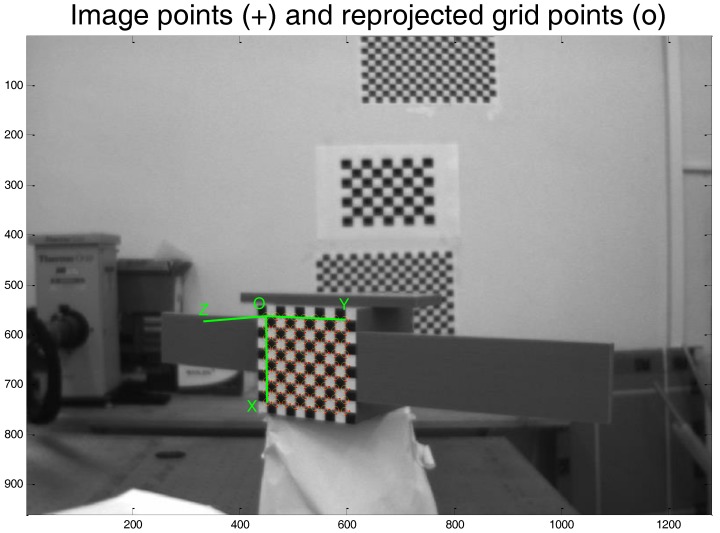
Example of corner extraction from a target image using the Camera Calibration Toolbox for MATLAB.

Standard solutions are adopted for both the corner extraction and the P*n*P solver. Specifically, they are implemented using the “Extrinsic Camera Parameters calculator” of the Camera Calibration Toolbox for MATLAB (for instance, one example of corner extraction using this toolbox is given by Figure 6). At the end of this process, according to the notation provided at the beginning of this section, an accurate estimate of the camera-to-target relative position vector (*T_C-T_^C^*) and rotation matrix ($\underline{\underline{R}}$*_C-T_*) is obtained. Although a direct measure of the pose accuracy level is not available, an indirect information about the quality of the pose estimate is given by the standard deviation of the corners’ re-projection error.

## 4. LIDAR/Camera Relative Calibration

The process of estimating the parameters representing the extrinsic relative calibration between a 3D scanning LIDAR and a monocular camera mounted with a fixed configuration, is now presented. A fundamental requirement is that the two sensors must share a portion of their FOVs, which must be large enough to adequately include the calibration board. It is important to underline that planarity and edge linearity are the only geometrical requirements which the calibration board must met. This means that the support of the checkerboard must be quadrangular but it may be non-rectangular. Also, the checkerboard may not be perfectly attached at the centre of its support. The camera-to-LIDAR relative rotation matrix ($\underline{\underline{R}}$*_C-L_*) and position vector (*T_L-C_^L^*) are determined in two subsequent steps.

### 4.1. Relative Attitude Estimation

The relative attitude between two reference frames can be derived from a set of vector measurements by using either deterministic (e.g., TRIAD) or probabilistic approaches (e.g., QUEST) [40]. Specifically, these methods require that the unit vectors corresponding to two or more independent (i.e., non-parallel) directions are determined in both the reference frames. In the case of interest, a set of images and point clouds are acquired by the camera and the LIDAR by placing the calibration board at *N* ≥ 2 distinct locations, arbitrarily spread in the portion of the FOV shared by the two sensors. These data are then processed to extract the directions of the outer normal to the planar grid in both L (*n^L^*) and C (*n^C^*).

With regards to the monocular images, the extrinsic calibration function of the MATLAB camera calibration toolbox can be used to estimate the pose of the grid with respect to the camera, i.e., a rotation matrix ($\underline{\underline{R}}$*_G-C_*) and a position vector (*T_C-G_^C^*), by solving the P*n*P problem. Since the third axis of the grid reference frame (G) identifies the normal to the checkerboard (see Section 2), *n^C^* is equal to the third column of $\underline{\underline{R}}$*_G-C_*. As for the monocular pose determination process, a measure of the accuracy in the estimation of *n^C^* is given by the standard deviation of the re-projection error of the corners extracted from the grid (*ε_rp_*), which is evaluated along the horizontal and vertical direction on the image plane using Equation (4):

(4) $$\begin{array}{l} {\varepsilon_{rp}|}_{x}=\sqrt{\frac{1}{N_{c}-1}{\sum_{i=1}^{N_{c}}\left|\varepsilon_{i,x}-\frac{1}{N_{c}}\sum_{i=1}^{N_{c}}\varepsilon_{i,x}\right|}^{2}}\\ {\varepsilon_{rp}|}_{y}=\sqrt{\frac{1}{N_{c}-1}{\sum_{i=1}^{N_{c}}\left|\varepsilon_{i,y}-\frac{N_{c}}{N_{c}}\sum_{i=1}^{N_{c}}\varepsilon_{i,y}\right|}^{2}} \end{array}$$

*ε_i,x_* and *ε_i,y_* are the horizontal and vertical components of the re-projection error for the *i*th corner, while *N_c_* is the total number of corners extracted from the imaged checkerboard (i.e., 247).

**Figure 7:**
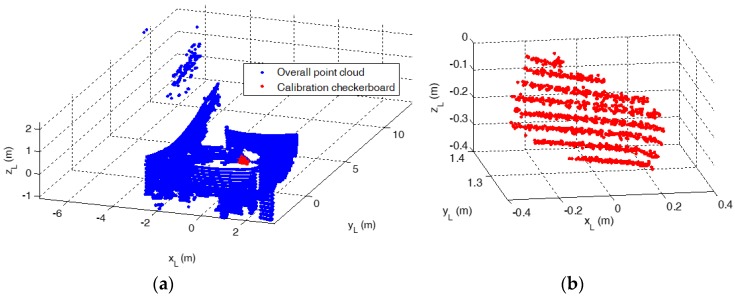
Point-cloud view of the calibration environment (**a**); Segmented calibration board (**b**).

Due to the panoramic FOV of the VLP-16 (i.e., 360° in azimuth), the LIDAR measurements corresponding to the calibration board must be separated from the background. This is done exploiting a coarse knowledge of the location of the checkerboard as well as the range discontinuity between the points of interest and the rest of the calibration environment. An example of result of this operation is shown by Figure 7. After this segmentation, *n^L^* can be estimated by performing plane fitting within the MATLAB curve fitting toolbox. Specifically, the least absolute residuals (LAR) option is selected, which fits a plane to the measured dataset by minimizing the absolute difference of the residuals, rather than the squared differences. The minimization is carried out by means of the Levenberg-Marquardt algorithm [41]. The root mean squared error (RMSE) of the measured points with respect to the fitted plane is used to indicate the accuracy level in the estimation of *n^L^*.

At this point, the camera-to-LIDAR relative rotation matrix ($\underline{\underline{R}}$*_C-L_*) is determined by applying the QUEST algorithm. Given the relation between the estimated values of *n^C^* and *n^L^*:

(5) $${\underline{\underline{R}}}_{C-L}{\underline{n}}_{C}{}^{i}={\underline{n}}_{L}{}^{i},\begin{array}{ccc} & & i=1,2,\ldots N \end{array}$$

this technique allows estimating the rotation matrix by optimizing the Wahba’s Loss function (*W*) shown by Equation (6):

(6) $$W=\frac{1}{2}\sum_{i=1}^{N}a_{i}{\left|{\underline{n}}_{L}{}^{i}-{\underline{\underline{R}}}_{C-L}{\underline{n}}_{C}{}^{i}\right|}^{2}$$

where *a_i_* is a weight term inversely proportional to the RMSE associated to plane fitting:

(7) $$a_{i}=\frac{\frac{1}{{\mathrm{RMSE}}_{i}}}{\sum_{i=1}^{N}\left(\frac{1}{{\mathrm{RMSE}}_{i}}\right)}$$

Clearly, since the QUEST algorithm estimates the relative attitude in the form of an optimal quaternion (*q_opt_*), a proper conversion is required to get the correspondent rotation matrix [38].

**Figure 8:**
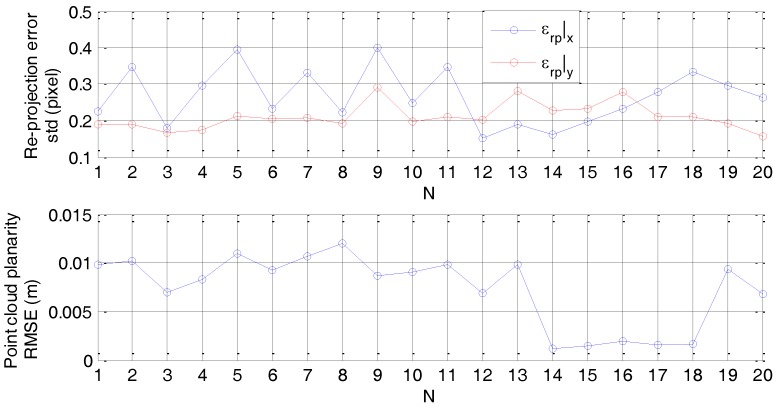
Relative calibration input data quality assessment. Camera data: standard deviation of the corner re-projection error for each of the recorded images of the calibration target (**up**). LIDAR data: RMSE from plane fitting for each of the recorded point cloud of the calibration target (**down**). Horizontal axis reports the number of acquired datasets.

The camera-to-LIDAR relative attitude is estimated using a set of 20 images and point clouds of the calibration board. A summary of the accuracy attained for the determination of *n^C^* and *n^L^* is given by Figure 8. Specifically, it shows that the average value of *ε_rp_|_x_* and *ε_rp_|_y_* is well below half of a pixel, i.e., 0.27 and 0.21 pixels, respectively. Instead, the average level of the RMSE from plane fitting is around 7 mm.

Finally, for the sake of clarity, the estimated ${\underline{\underline{R}}}_{C-L}$ is expressed in the form of a 321 sequence of Euler angles (EA). The first two rotations are nearly zero, i.e., 0.91° and 0.97°, respectively, while the third rotation is 90.75°. This result is consistent with the mounting configuration for the two sensors (i.e., *y^L^* and *z^C^* are nearly co-aligned). Indeed, with the adopted hardware (i.e., considering the manufacturing tolerances of the bracket) and based on previously shown axes’ conventions, the reference relative orientation between the LIDAR and the camera is identified by the 321 EA sequence (0°, 0°, 90°), with an uncertainty of the order of 1° for each EA.

### 4.2. Relative Position Estimation

The process of determining *T_L-C_^L^* is started by selecting a subset of *M* (*≤N*) of the point clouds used in the previous step of the calibration procedure (after segmentation with respect to the rest of the calibration environment). The criterion used for selection is that the point cloud shall contain at least one LIDAR stripe (i.e., subset of measurements collected by the same laser/detector couple of the VLP-16) intersecting non-parallel sides of the calibration board. Under this condition, two independent estimates of *T_L-C_^L^* can be obtained for each LIDAR stripe.

**Figure 9:**
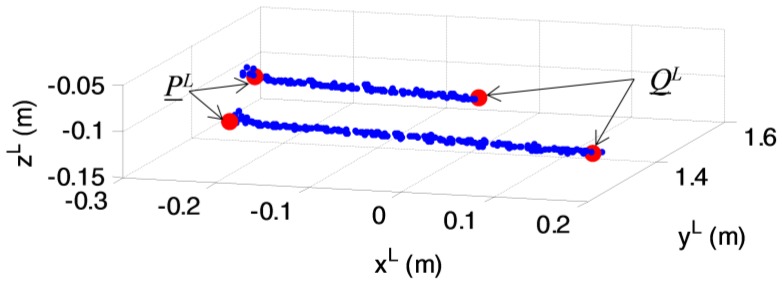
Extraction of the interception points between the LIDAR stripes and the sides of the checkerboard.

First, the stripes are processed applying a line fitting algorithm which aims at extracting the points in which they intersect the sides of the calibration board. The result of this operation is shown by Figure 9, where *P^L^* and *Q^L^* are the position vectors of the two ends of the LIDAR segment. Clearly, the vector corresponding to this segment (*PQ*) can also be determined in both L and C, as shown by Equation (8):

(8) $$\begin{array}{l} {\underline{PQ}}^{L}={\underline{P}}^{L}-{\underline{Q}}^{L}\\ {\underline{PQ}}^{C}={\left({\underline{\underline{R}}}_{C-L}\right)}^{t}{\underline{PQ}}^{L} \end{array}$$

**Figure 10:**
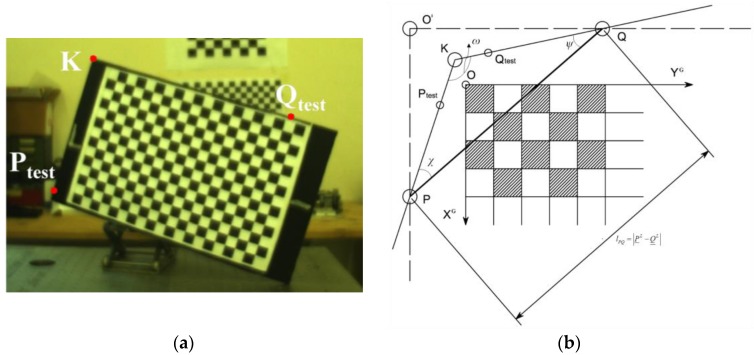
Auxiliary points extracted from the image of the calibration board (**a**). Geometry of the relative position determination approach (**b**).

At this point, the goal of the relative calibration process is to estimate the position of *P* and *Q* in C (i.e., *P^C^* and *Q^C^*) so that *T_L-C_^L^* can be computed by means of a vector composition. To this aim, three points must be manually selected on the corresponding image as shown by Figure 10a. One point (*K*) is the corner of the calibration board shared by the two sides intercepted by the LIDAR stripe. The other two points (*P_test_* and *Q_test_*) can be freely selected along those two sides. Thanks to the knowledge of the intrinsic parameters of the camera, the pixel location on the image plane can be used to determine the unit vectors pointing at *K*, *P_test_* and *Q_test_* in C. Consequently, the corresponding position vectors (i.e., *K^C^*, *P_test_^C^* and *Q_test_^C^*) can be estimated as the intersection between the directions of these unit vectors with the plane of the checkerboard (which is known from the estimation of the camera pose with respect to the grid). Given these inputs, the determination of *P^C^* and *Q^C^* is based on the geometry depicted by Figure 10b, where the deviation from perpendicularity of the two sides of the support hosting the checkerboard is enhanced for the sake of clarity of the description.

The main idea is to solve the triangle composed of the points *K*, *Q*, *P.* Specifically, the three inner angles of the triangle can be computed using Equation (9):

(9) $$\begin{array}{l} \psi =\cos^{-1}\left(\left(\frac{{\underline{K}}^{C}-{\underline{Q}}_{test}{}^{C}}{\left|{\underline{K}}^{C}-{\underline{Q}}_{test}{}^{C}\right|}\right)\cdot \frac{{\underline{PQ}}^{C}}{\left|{\underline{PQ}}^{C}\right|}\right)\\ \chi =\cos^{-1}\left(\left(-\frac{{\underline{K}}^{C}-{\underline{P}}_{test}{}^{C}}{\left|{\underline{K}}^{C}-{\underline{P}}_{test}{}^{C}\right|}\right)\cdot \frac{{\underline{PQ}}^{C}}{\left|{\underline{PQ}}^{C}\right|}\right)\\ \omega =\cos^{-1}\left(\left(\frac{{\underline{K}}^{C}-{\underline{P}}_{test}{}^{C}}{\left|{\underline{K}}^{C}-{\underline{P}}_{test}{}^{C}\right|}\right)\cdot \left(\frac{{\underline{K}}^{C}-{\underline{Q}}_{test}{}^{C}}{\left|{\underline{K}}^{C}-{\underline{Q}}_{test}{}^{C}\right|}\right)\right) \end{array}$$

Since the three inner angles as well as the length of one side (*l_PQ_*, obtained as the norm of *P^L^* − *Q^L^*) are known, the length of the other two sides (i.e., *l_KP_* and *l_KQ_*) can be determined using the law of sines. Consequently, *P^C^* and *Q^C^* can be estimated using Equation (10):

(10) $$\begin{array}{l} {\underline{P}}^{C}={\underline{K}}^{C}+{\underline{KP}}^{C}={\underline{K}}^{C}+l_{KP}\left(\frac{{\underline{K}}^{C}-{\underline{P}}_{test}{}^{C}}{\left|{\underline{K}}^{C}-{\underline{P}}_{test}{}^{C}\right|}\right)\\ {\underline{Q}}^{C}={\underline{K}}^{C}+{\underline{KQ}}^{C}={\underline{K}}^{C}+l_{KQ}\left(\frac{{\underline{K}}^{C}-{\underline{Q}}_{test}{}^{C}}{\left|{\underline{K}}^{C}-{\underline{Q}}_{test}{}^{C}\right|}\right) \end{array}$$

At this point, a vector composition allows computing *T_L-C_^L^* as shown by Equation (11) using both *P* and *Q*:

(11) $$\begin{array}{l} {{\underline{T}}_{L-C}{}^{L}|}_{P}={\underline{P}}^{L}-{\underline{\underline{R}}}_{C-L}{\underline{P}}^{C}\\ {{\underline{T}}_{L-C}{}^{L}|}_{Q}={\underline{Q}}^{L}-{\underline{\underline{R}}}_{C-L}{\underline{Q}}^{C} \end{array}$$

The results of this calibration procedure are collected in Table 1.

A final estimate of the LIDAR-to-camera relative position vector is obtained as the mean of the independent estimates corresponding to the *P* and *Q* points of each appropriate LIDAR stripe (i.e., suitable for applying the calibration procedure according to the criterion mentioned at the beginning of this sub-section) composing the *M* (i.e., 7) selected point clouds. This operation is critical to reduce the noise from the individual vector computation, and the corresponding uncertainty (1 σ) is also indicated. Again, this estimate is consistent with the mounting configuration and the knowledge of sensors/setup dimensions. Overall, the LIDAR/camera extrinsic calibration process has led to sub-degree and 1-centimeter order accuracies in the relative attitude and position, respectively.

## 5. Pose Determination Experimental Results

Once an image and a point cloud of the target are acquired, the algorithms presented in Section 3 are used to get a high-accuracy, monocular estimate of the pose of the target with respect to the camera (i.e., *T_C-T_^C^|_m_*, ${\underline{\underline{R}}}_{C-T}{|}_{m}$) and to initialize the pose of the target with respect to the LIDAR (i.e., *T_L-T_^L^|_l,init_* and ${\underline{\underline{R}}}_{T-L}{|}_{l,init}$). Clearly, the accuracy levels attained by the LIDAR-based pose initialization techniques is much lower than the monocular pose estimate. However, the initial pose acquisition, even if coarse, is considered successful if it falls in the field of convergence of the tracking algorithm. So, the initial pose estimate is refined using a NN implementation of the Iterative Closest Point algorithm (ICP) [42]. Finally, the refined LIDAR-based pose estimate (i.e., *T_L-T_^L^|_l,ref_* and ${\underline{\underline{R}}}_{T-L}{|}_{l,ref}$) is converted to the camera using the parameters of the LIDAR/camera extrinsic calibration, as shown by Equation (12):

(12) $$\begin{array}{l} {{\underline{T}}_{C-T}{}^{C}|}_{l,ref}={\left({\underline{\underline{R}}}_{C-L}\right)}^{t}\left({{\underline{T}}_{L-T}{}^{L}|}_{l,ref}-{\underline{T}}_{L-C}{}^{L}\right)\\ {{\underline{\underline{R}}}_{C-T}|}_{l,ref}={\left({{\underline{\underline{R}}}_{T-L}|}_{l,ref}\right)}^{t}\left({\underline{\underline{R}}}_{C-L}\right) \end{array}$$

**Figure 11:**
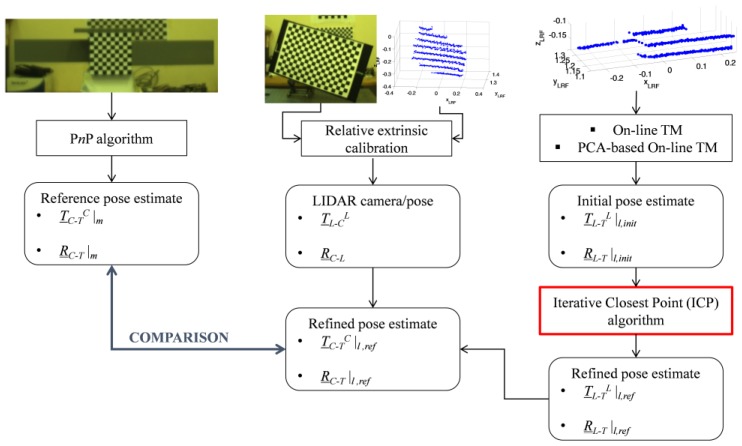
Detailed test strategy. The location at which the ICP algorithm is introduced to refine the LIDAR-based pose estimate is highlighted in red. Please note that the relative extrinsic calibration is carried out off-line with respect to the data acquisitions and algorithm runs for pose determination.

For the sake of clarity, the performance evaluation strategy described above is summarized by Figure 11, which also contains examples of images and point cloud of the target and the calibration board.

**Figure 12:**
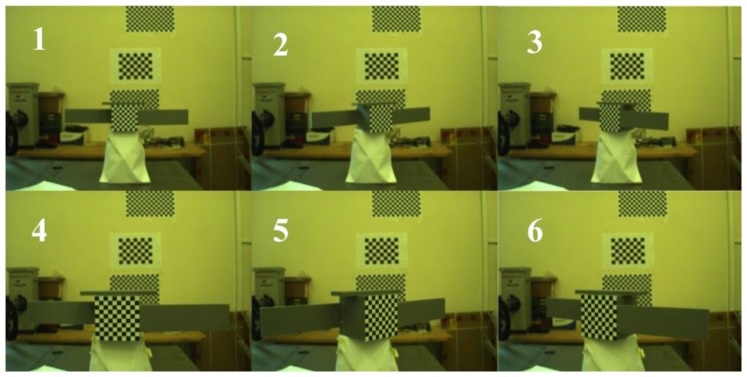
Monocular images for the analyzed test cases.

Six test cases (TC) are analyzed, for which the images acquired by the AVT camera are shown in Figure 12. The TC pairs {1, 4}, {2, 5}, {3, 6} are characterized by the same attitude conditions but different sensor-target distances, i.e., 1.1 m and 0.7 m for the former and the latter TC, respectively, in each pair. The monocular-based pose estimation approach presented in Section 3.2 is applied for each of these images, and *ε_rp_* is approximately 0.1 pixel both in the horizontal and vertical direction on the focal plane. Given that the AVT camera IFOV is 0.04°, and, according to open literature results [11], in the considered interval of distances, the application of standard P*n*P solutions to recognized cooperative markers allows getting sub-millimeter and cents-of-degree accuracy in relative position and attitude, respectively. This implies that, despite the absence of truth-pose data, *T_C-T_^C^|_m_* and ${\underline{\underline{R}}}_{C-T}{|}_{m}$ can be considered valid benchmarks to assess the absolute performance of the LIDAR-based uncooperative pose estimation algorithms presented in Section 3.1.

The metrics selected for performance evaluation are defined as follows. The difference between the Euclidean norms of *T_C-T_^C^|_m_* and *T_C-T_^C^|_l,ref_* (*|T_ERR_|*) is adopted for relative position. The equivalent Euler angle (*φ_ERR_*) corresponding to the quaternion error (*q_ERR_*) between ${\underline{\underline{R}}}_{C-T}{|}_{m}$ and ${\underline{\underline{R}}}_{C-T}{|}_{l,ref}$ is used for relative attitude. The pose estimation errors for the on-line TM and the PCA-based on-line TM algorithms after the ICP refinement are collected in Table 2.

The value of *|T_ERR_|* is indicated only for the former technique since they both exhibit centimeter-level relative position errors, with differences in the order of 10^−5^ m. This occurs because the ICP algorithm exploits the initial position provided by the centroiding approach using (1) for both the on-line TM and the PCA-based on-line TM. Also, no substantial effect on accuracy can be noticed due to the different distance from the target in each TC couple.

With regards to the comparison between the two analyzed techniques, the on-line database generation is carried out by setting *Δ* to 30° for both the cases. This leads to a database composed of 1183 and 13 templates for the on-line-TM and the PCA-based on-line TM, respectively. Consequently, the use of the PCA to directly solve for two of the three rotational degrees of freedom allows to reduce the computational load of one order of magnitude. Indeed, the computational time (on a commercial desktop equipped with an Intel™ i7 CPU at 3.4 GHz) is around 10 s and 0.5 s for the two approaches. On the other hand, the on-line TM is more robust against variable pose conditions also because its implementation is invariant with respect to the object shape. The PCA-based algorithm, instead, is not applicable if the target does not have an elongated shape. Moreover, also in the case of elongated objects, the PCA-based on-line TM may be negatively affected by self-occlusions which do not allow to extract information about the target principal directions from the collected data.

The higher level of robustness of TM with respect to the PCA-based algorithm can be noticed if the attitude error is compared. Indeed, even if both techniques exhibit very similar attitude errors, of a few degrees, it is possible to notice that, for the TC-5, the error provided by the PCA-based algorithm gets larger (around 15°) than average performance. However, this error can still be accepted since it is well below the adopted value of *Δ*, thus tacking full advantage from the reduced computational load offered by the PCA-based algorithm*.* Indeed, numerical simulations demonstrated that such an initial pose error is suitable for allowing the ICP algorithm to attain sub-degree and sub-cm accuracy during tracking [16,18]. Clearly, this point shall be further analyzed by performing also dynamic acquisitions using an updated/improved version of this experimental setup.

## 6. Conclusions

This paper presented a strategy for hardware-in-the-loop performance assessment of algorithms designed to estimate the relative position and attitude (pose) of uncooperative, known targets by processing range measurements collected using active LIDAR systems. The pose determination techniques, originally developed by the authors and presented in previous papers, rely on concepts like correlation-based template matching and principal component analysis and did not require cooperative markers but only the knowledge of the target geometry.

The experimental setup, firmly installed on an optical bench, included a monocular camera and a scanning LIDAR mounted according to a fixed configuration in order to share a large portion of their respective fields of view. The two sensors were used to simultaneously collect images and point clouds of the same target, i.e., a scaled-replica of a satellite mock-up. A state-of-the-art solution of the perspective-n-point algorithm, which exploited the recognition of cooperative markers installed on the target surface, provided a high-accuracy estimate of the target pose with respect to the camera. This monocular pose estimate was characterized by sub-degree and sub-millimeter accuracies in relative attitude and position, respectively, in the considered range of target/sensor distances. Thus, it is used as a benchmark to determine the absolute pose determination accuracy of the LIDAR-based algorithms. Hence, a critical point of this work was the necessity to obtain an accurate estimate of the relative extrinsic calibration between the camera and the LIDAR. To this aim an original approach was proposed. It relied on a semi-analytic, non-iterative procedure which does not need homologous points to be directly searched in the scene, but rather it required relaxed geometrical constraints on the calibration target to be satisfied. The relative attitude and position between the camera and the LIDAR were estimated with sub-degree and sub-centimeter accuracies, respectively. Given the result of the relative calibration and the monocular pose estimate as benchmark, the investigated LIDAR-based algorithms were able to successfully initialize the pose of a non-cooperative target with centimeter and a few degree accuracies in relative position and attitude, respectively. Future works will be addressed to update the experimental setup, e.g., by the design of a specific support for the target, in order to significantly enlarge the range of poses that can be reproduced. Also, motion control systems will be introduced to perform dynamic tests.

Finally, it is worth outlining that the proposed method for LIDAR/camera relative calibration could be relevant to a wider range of autonomous vehicles (marine, terrestrial, aerial, space) which use a scanning LIDAR and a camera partially sharing their field-of-views for navigation or situational awareness purposes.

## Figures and Tables

**Table 1:** Results of LIDAR-to-camera relative position estimation process. The ID of the LIDAR stripe identifies a specific elevation angle (detailed information can be found in [36]).

Point Cloud ID	LIDAR Stripe ID	*T_L-C_^L^*|*_P_* (m)			*T_L-C_^L^*|*_Q_* (m)		
		*x^L^*	*y^L^*	*z^L^*	*x^L^*	*y^L^*	*z^L^*
1	10	0.01	0.09	−0.05	0.00	0.07	−0.05
1	12	0.02	0.09	−0.06	0.01	0.08	−0.06
2	10	0.02	0.07	−0.06	0.01	0.06	−0.06
2	12	0.01	0.08	−0.06	0.01	0.07	−0.06
8	10	0.01	0.07	−0.06	0.00	0.06	−0.06
8	12	0.01	0.08	−0.05	0.01	0.08	−0.05
8	14	0.01	0.07	−0.04	0.00	0.04	−0.05
10	12	0.00	0.08	−0.05	0.00	0.08	−0.05
10	14	0.00	0.07	−0.06	0.00	0.07	−0.06
11	12	0.00	0.07	−0.06	0.00	0.08	−0.06
11	14	0.00	0.08	−0.05	0.01	0.07	−0.05
14	5	0.00	0.06	−0.05	0.00	0.07	−0.05
19	1	0.00	0.09	−0.05	−0.01	0.07	−0.05
19	12	0.00	0.08	−0.06	0.00	0.07	−0.06
19	14	0.01	0.07	−0.05	0.01	0.05	−0.05
**Mean ± 1 σ**		${\underline{T}}_{L-C}{}^{L}=\left[\begin{array}{c} x^{L}\\ y^{L}\\ z^{L} \end{array}\right]=\left[\begin{array}{c} 0.005\pm 0.006\\ 0.072\pm 0.011\\ -0.054\pm 0.003 \end{array}\right](m)$					

**Table 2:** Accuracy level of the LIDAR-based pose solution for the analyzed test cases.

Test Cases	LIDAR-Based Pose Solver	*|T_ERR_|* (m)	*φ_ERR_* (°)	LIDAR-Based Pose Solver	*φ_ERR_* (°)
1	On-line TM + NN-based ICP	0.004	1.3	PCA-based on-line TM + NN-based ICP	1.7
2		0.011	5.5		5.1
3		0.014	3.6		3.2
4		0.005	1.3		1.4
5		0.008	4.6		15.1
6		0.010	2.9		2.8
